# Diversity of Circulating NKT Cells in Defense against Carbapenem-Resistant Klebsiella Pneumoniae Infection

**DOI:** 10.3390/jpm12122025

**Published:** 2022-12-07

**Authors:** Yidi Wang, Feng Zang, Xiangyu Ye, Zhanjie Li, Wenhao Zhu, Xiaoxiao Cao, Xuehong Cai, Xinyan Ma, Lei Xu, Yongxiang Zhang, Liqing Bi, Rongbin Yu, Peng Huang

**Affiliations:** 1 Department of Epidemiology, Center for Global Health, School of Public Health, Nanjing Medical University, Nanjing 210000, China; 2 Department of Infection Control, The First Affiliated Hospital of Nanjing Medical University (Jiangsu Province Hospital), Nanjing 210000, China; 3 Department of Neurosurgery ICU, The First Affiliated Hospital of Nanjing Medical University (Jiangsu Province Hospital), Nanjing 210000, China

**Keywords:** carbapenem-resistant Klebsiella pneumoniae, natural killer T cells, host immunity, scRNA-seq

## Abstract

Nosocomial infection caused by carbapenem-resistant Klebsiella pneumonia (CRKP) infection has become a global public health problem. Human NK and NKT cells in peripheral immune responses are recognized as occupying a critical role in anti-bacterial immunity. Through performed scRNA-seq on serial peripheral blood samples from 3 patients with CRKP undergoing colonization, infection, and recovery conditions, we were able to described the immune responses of NK and NKT cells during CRKP infection and identified a mechanism that could contribute to CRKP clearance. The central player of CRKP infection process appears to be the NKT subset and CD56hiNKT subset which maintained immune competence during CRKP colonization. With time, CRKP leads to the loss of NK and CD160hiNKT cells in peripheral blood, resulting in suppressed immune responses and increased susceptibility to opportunistic infection. In summary, our study identified a possible mechanism for the CRKP invasion and to decipher the clues behind the host immune response that influences CRKP infection pathogenesis.

## 1. Introduction

In recent years, carbapenem-resistant Klebsiella pneumoniae (CRKP) infection has become a global public health problem [1,2]. CRKP causes a greater than 45.9% mortality rate of nosocomial infection, even with antibiotic treatment [3,4]. A great number of patients with CRKP colonization developed CRKP infection characterized by an excessive inflammatory response, which plays a major role in later pneumonia, bacteremia, liver abscess, and other diseases [5,6,7]. The host immune system plays an important role in the occurrence of CRKP infection [2,8]. However, the mechanisms that regulate the clearance of CRKP infection and the immune reactions of post-colonization are incompletely defined. Therefore, a better definition of immune cells is urgently required to support the exploration of newer host-directed therapies to combat CRKP infection.

Studies have reported that natural killer T (NKT) cells can mediate a pivotal role in the clearance of bacteria by secreting cytokines and chemokines followed T cell receptors (TCRs) engagement and regulating the distribution of NK cell receptors [9,10]. In addition, NK cells have been reported as the dominant cellular source for NKT-induced cytokines, which help to clear secondary Candida infection in sepsis patients [11]. With the development of biotechnology and in-depth research on NKT cells, NKT types have been further subdivided, and the functional characteristics of different subtypes in the occurrence and development of diseases have been gradually clarified. Each NKT subset could potentially and variably contribute to modulating the antigen-specific immune response [12,13]. The contribution of NKT cells to CRKP infection pathogenesis is not yet known. Hence, detailed studies on NKT cells compartments and functional changes are required for CRKP infection.

Our objective was to explore blood NKT cell heterogeneity during CRKP infection. ScRNA-seq is a powerful technique for discovering NKT cells transcriptional diversity. We performed scRNA-seq on peripheral blood from CRKP colonization, infection, and recovery patients, which helps to elucidate host–pathogen interactions evolving over time and reveal the underlying cellular and molecular mechanisms of CRKP infection. The results described the immune responses of NK and NKT cells during CRKP infection and identified a mechanism that could contribute to CRKP clearance.

## 2. Materials and Methods

### 2.1. Clinical Samples Collection

Whole blood samples were collected from patients diagnosed with CRKP colonization, infection. and healthy control admitted to The First Affiliated Hospital with Nanjing Medical University between June 2021 and July 2022. The cohort enrolled adult patients with a diagnose of CRKP colonization. We collected the blood samples from patients at the time of enrollment, CRKP infection, and after anti-CRKP treatment. Diagnosis of CRKP colonization and infection were based on the clinical symptoms, chest radiography, and antimicrobial susceptibility tests by the clinician. Matched samples (*n* = 9) from three patients under CRKP colonization, infection, and recovery conditions and one healthy control sample were recruited to perform scRNA-seq experiments. This study was approved by the institutional review board, and informed written consent was obtained from each participant.

### 2.2. Single-Cell RNA Sequencing

For scRNA-seq, whole blood samples were diluted with equal volumes of phosphate buffered saline, and then peripheral blood mononuclear cells (PBMCs) were separated following density gradient centrifugation methods using Ficoll. For each sample, the viability of PBMCs exceeded 90%. The samples were then loaded onto the 10X Genomics platform to create a barcoded cDNA library for individual cells. Single-cell libraries were sequenced on an Illumina NovaSeq 6000 system, targeting ∼300 M raw reads per sample (∼60,000 raw reads per cell).

### 2.3. Single-Cell Data Processing

Sequence data from 10X Genomics were processed using Cell Ranger (v.6.1.2, 10X Genomics, Pleasanton, CA, USA) and matched to the GRCh38 reference genome to generate filtered gene-barcode matrices. Then, we applied the quality control, data normalization, and scaling workflows to count the matrices using *Seurat* (v.4.1.1, Gribov et al., Augsburg, Germany) [14]. First, we removed cells expressing fewer than 300 genes or mitochondrial gene content >30% of the total UMI count and excluded genes expressed in fewer than 3 cells [15]. We defined NK and NKT cells by classical markers (CD3E and KLRF1). With this procedure, 16,021 cells were retained for further analytical processing. Next, gene expression matrices were normalized by the global-scaling normalization method *LogNormalize* to the total cellular and mitochondrial read counts using a linear regression model. After data normalization, we identified the top 3000 highly variable genes using the *FindVariableFeatures* function. Subsequently, we used principal component analysis (PCA) with variable genes to perform the linear dimensional reduction after a linear transformation performed by *ScaleData*. The top 50 principal components were selected as the input for uniform manifold approximation and projection (UMAP). Cell clustering was done using the *FindClusters* function, and the cell types were annotated using a combination of the R package *FindAllMarkers*. A marker gene was defined as one that was expressed in at least 10% of cells in the more abundant cell type and with log-transformed fold change >0.25 and *p* value < 0.01 in that cell type under comparison [16].

Differential expression genes (adjusted *p* value < 0.05 and |Log2FC| > 0.25) were put into the *clusterProfiler* (v.4.2.2, Yu et al., Guangzhou, China) workflow for kyoto encyclopedia of genes and genomes (KEGG) and gene ontology (GO) analysis (https://david.ncifcrf.gov/) (accessed on 10 August 2022) [17]. Pathways with adjusted *p* value < 0.05 were considered significantly enriched. To illustrate the functional properties of different cell types, defense response score was defined as the mean expression levels of function-related genes, DEFENSE RESPONSE (GO: GO:0006952), by using the *AddModuleScore* function. Gene set variation analysis (GSVA) was performed on the 50 hallmark pathways annotated in the molecular signature database (MSigDB v7.2, http://software.broadinstitute.org/gsea/msigdb/index.jsp) (accessed on 19 June 2022) using *GSVA* (v.1.42.0, Hänzelmann et al., Catalonia, Spain) [18]. The gene expression levels of transcription factor (TFs) were defined using the lightning-fast python implementation of the SCENIC pipeline, pySCENIC (v.0.1.2, Van de Sande et al., Leuven, Belgium) [19].

### 2.4. RNA Velocity Analyses

The RNA velocity analysis was used to estimate the future developmental directionality of each cell that relied on mRNA abundance (mature spliced/nascent unspliced transcripts) [20]. Based on the velocyto workflow, annotation of mRNA abundance was performed using the Python script velocyto.py on the Cell Ranger output folder. The RNA velocity was generated using a gene-relative model with k-nearest neighbor cell pooling. Velocity fields were projected onto the pseudotime space generated by Monocle 3.

### 2.5. Time Dependent Transcriptional Analysis

To identify time dependent transcriptional programs in CRKP infection, we applied the *Mfuzz* (v.2.54.0, Kumar and M, Berlin, Germany) function, which subjected the activated candidate genes of the NKT subset to unsupervised clustering by a fuzzy c-means algorithm [21]. We calculated the average gene expression for each stage. Then, we added 0.000001 to each gene expression matrix to avoid 0, which is not acceptable for Mfuzz. After that, we performed the *fifilter.std* function to standardize the expression values of genes and estimated an optimal setting of the fuzzifier with the *mestimate* function according to the tutorial. Six different expression programs were clustered for future analysis.

### 2.6. Cell–Cell Communication Analysis

We applied the CellChat workflow to assess putative interactions between different cell types (NK, NKT, CD56hiNKT, and CD160hiNKT). The process and description of CellChat analysis are detailed at https://github.com/sqjin/CellChat (accessed on 10 October 2022). Significant interactions between cell types were defined as those with adjusted *p* value < 0.05. For downstream analysis, we focused on interactions between the NK and NKT subset.

### 2.7. Statistical Analysis

All statistical analyses were performed in R (version 4.1.0, R core team, Vienna, Austrilia). Tukey’s HSD post-test and ANOVA were used to make comparisons between multiple groups. Two-tailed statistical tests were conducted, and adjusted *p* value < 0.05 was considered statistically significant: * *p* < 0.05, ** *p* < 0.01, *** *p* < 0.001, **** *p* < 0.0001.

## 3. Results

### 3.1. NK and NKT Subset Distribution in Patients during CRKP Infection

**Figure 1:**
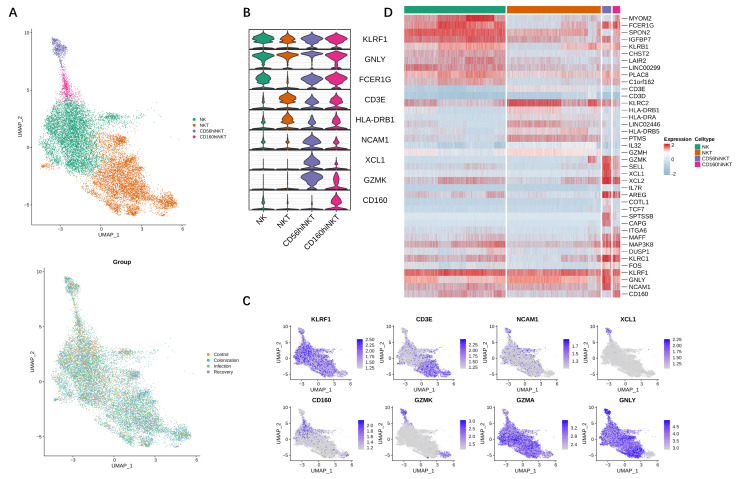
Circulating NK and NKT subset diversity during CRKP infection. (**A**) The UMAP visualization of individual cells from 10 PBMC samples taken from healthy donors (*n* = 1) and patients across CRKP colonization (*n* = 3), infection (*n* = 3), and recovery (*n* = 3). Each dot corresponds to a single cell, colored according to cell type (**upper**) and CRKP infection conditions (**lower**). (**B**) Violin plots showing the expression distribution of selected canonical cell markers in the four clusters. The rows represent selected marker genes and the columns represent clusters with the same color as in (**A**). (**C**) Canonical cell markers were used to label clusters by cell identity as represented in (**A**). Data are colored according to expression levels, and the legend is labeled in log scale. (**D**) Gene expression heatmap in each cell cluster of (**A**).

In order to obtain a more detailed insight into CRKP-induced alterations of the NKT cell pool, we examined transcriptional changes of NKT cells in the PBMC by scRNA-seq analysis. NKT and NK cells were from one healthy control and nine matched samples under CRKP colonization, infection, and recovery conditions. A total of 16,021 cells—1269 cells from the healthy control, 5206 cells from CRKP colonization patients, 5282 cells from CRKP infection patients, and 4222 cells from CRKP recovery patients—were processed according to the quality control procedures noted in the “Methods” section. The number of read counts and unique molecular identifiers detected per cell and the percentage of mitochondrial genes are shown in Supplemental Figure S1. Using unbiased clustering of cells, we captured the transcriptomes of the NK cluster (KLRF1, GNLY, FCER1G), NKT cluster (KLRF1, GNLY, CD3E, HLA-CRB1), CD56hiNTK cluster (KLRF1, GNLY, FCER1G, CD3E, HLA-CRB1, NCAM1, XCL1, GZMK), and CD160hiNKT cluster (KLRF1, GNLY, FCER1G, CD3E, HLA-CRB1, GZMK, CD160) based on the expression of canonical gene markers (Figure 1A–D). Each type of immune cells was observed across all four conditions (Figure 1A), suggesting that clustering was independent of sample-to-sample variability and infection condition.

### 3.2. Transcriptional Alterations of NK and NKT Subsets during CRKP Infection

**Figure 2:**
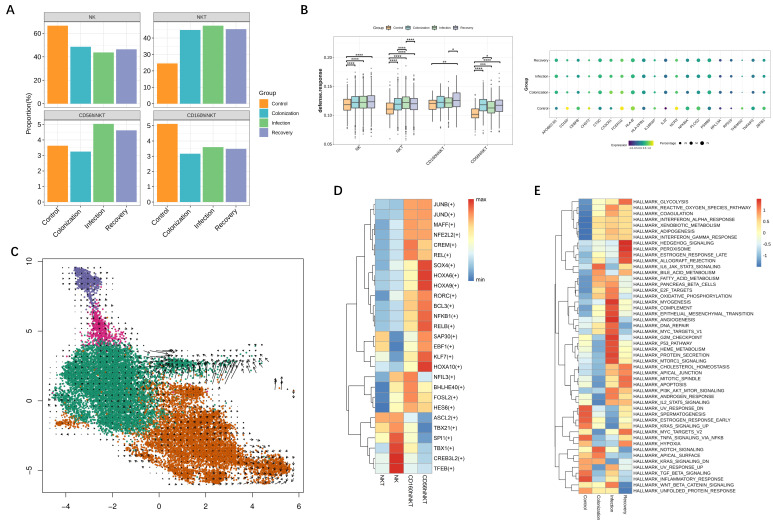
Circulating NK and NKT subset states during CRKP infection. (**A**) The cell type proportions across conditions. (**B**) Box plots of the response defense score across conditions. Conditions are shown in different colors. Horizontal lines represent median values; * *p*  <  0.05; ** *p*  <  0.01; *** *p*  <  0.001; **** *p*  <  0.0001; two-sided unpaired Dunn’s (Bonferroni) test. Dot plot showing expression of some genes associated with antibacterial response across conditions. The circle size and color, respectively, indicating the percentage and expression levels of cells expressing pathway-associated genes under each condition. (**C**) Potential developmental trajectory of NK and NKT clusters analyzed by RNA velocity. (**D**) TF analysis indicating the predicted regulators of each immune cell types. (**E**) GSVA analysis indicating the enriched Molecular Signatures Database hallmark gene sets of NK cluster across conditions.

We next assessed the distribution of NK and NKT subtypes with different CRKP infection states (Figure 2A). In patients with CRKP, both NK cell and CD160hiNKT cell abundance were roughly decreased to half of the control. The decline of circulating NK and CD160hiNKT cells indicates the immune response reduction of these clusters under CRKP infection. In addition, the defense response of NK and NKT subsets during CRKP infection development was evaluated with expression levels of the DEFENSE RESPONSE pathway in GO biological process terms. Compared with the control group, NK and NKT clusters showed upregulated defense response function across CRKP infection conditions. It is noteworthy that, compared with other clusters, CD56hiNKT cells exhibited the highest increase in the defense response after CRKP stimulation (Figure 2B). We then performed RNA velocity to better understand the developmental relationship between these cells. The resulting streamlines suggest NK cells potentially differentiate from immature cells in later stages (Figure 2C). Moreover, GSVA analysis across individual cell types using the MSigDB hallmark gene set showed function changes in NK cells during CRKP infection. NK cells in CRKP colonization patients significantly enriched pathways associated with IL6 (IL6_JAK_STAT3_SIGNALING) (Figure 2E). In addition, mTOR (MTORC1_SIGNALING), NF-kB related (P53_PATHWAY), and DNA_REPAIR pathways were enriched in NK cells under CRKP infection (Figure 2E).

**Figure 3:**
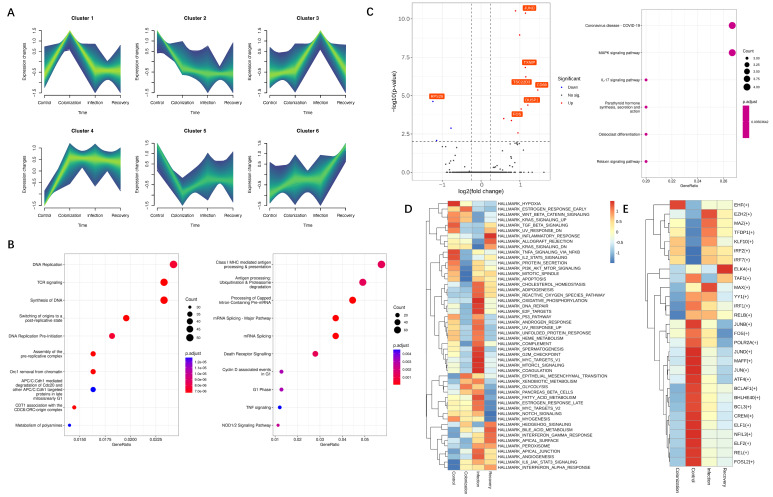
The function changes on NKT subsets during CRKP infection. (**A**) Genes contained in NKT clustered by their expression pattern along the progression of the disease by the mfuzz R package. The top 10 enriched biological processes in cluster 4 (**left**) and cluster 5 (**right**) of genes revealed by Reactome analysis. (**B**) Gene expression heatmap in each cell cluster of myeloid-cell subsets as defined in (**A**). (**C**) The left figure showed differentially expressed genes in the CD56hiNKT cluster from CRKP infection patients compared to healthy controls. Red dots represent genes upregulated in CRKP infection patients (adjusted *p* value < 0.01 and fold change (FC) ≥ 2), whereas blue dots represent downregulated genes in CRKP infection patients (adjusted *p* value < 0.01 and FC ≤ 0.5). Genes with log2(FC) ≥ 1.5 were labeled by gene symbols. The right figure shows enriched pathways of defined genes by KEGG analysis. (**D**) GSVA analysis indicating the enriched Molecular Signatures Database hallmark gene sets of the NKT cluster across conditions. (**E**) TF analysis indicating the predicted regulators of the NKT cluster across conditions.

The proportion of CD56hiNKT increased from 3.2% in the CRKP colonization group to 5.0% in the infection group (Figure 2A). We characterized the functions by applied Single-Cell Regulatory Network Inference and Clustering (SCENIC) analysis to correlate TFs with gene expression among NK and NKT types. Different TF expression patterns were identified for the cell types. The expression of genes regulated by NFKB1, RELB, and SAP30 was specifically upregulated in CD56hiNKT (Figure 2D). At the same time, we found that CD56hiNK cells showed activated IL-17 signaling pathway in CRKP infection patients, which was reported as a key mediator to bacterial infection (Figure 3C) [22,23].

Notably, the NKT cluster (24.6% in control group; 44.9% in colonization group; 47.6% in infection group; 45.5% in recovery group) was remarkably overrepresented in patients with CRKP (Figure 2A). We examined the gene transcription dynamics of the NKT subset during the CRKP infection process. We identified six time-dependent expression patterns and investigated their biological significance (Figure 3A and Figure S2A–D). Genes belonging to cluster 4 had increased expression levels across CRKP colonization, infection, and recovery. Enrichment analysis showed that their functions were enriched in TCR signaling and DNA replication pathways (Figure 3B). Cluster 5 of NKT was significantly decreased in CRKP colonization and then increased in infection and recovery patients (Figure 3A). The functions of these genes were enriched in class I MHC mediated antigen processing and presentation related pathway (Figure 3B). The transcriptional regulation of these genes in NKT cells is dynamic, which indicates the function of NKT changes during CRKP infection. GSVA analysis of NKT cells showed the enrichment of mTOR (MTORC1_SIGNALING) and OXIDATIVE_PHOSPHORYLATION pathways (Figure 3D). TF analysis identified that EZH2 and MAX mainly regulated NKT cells in CRKP infection conditions (Figure 3E).

### 3.3. The Regulatory Effect of NKT on NK Function during CRKP Infection

**Figure 4:**
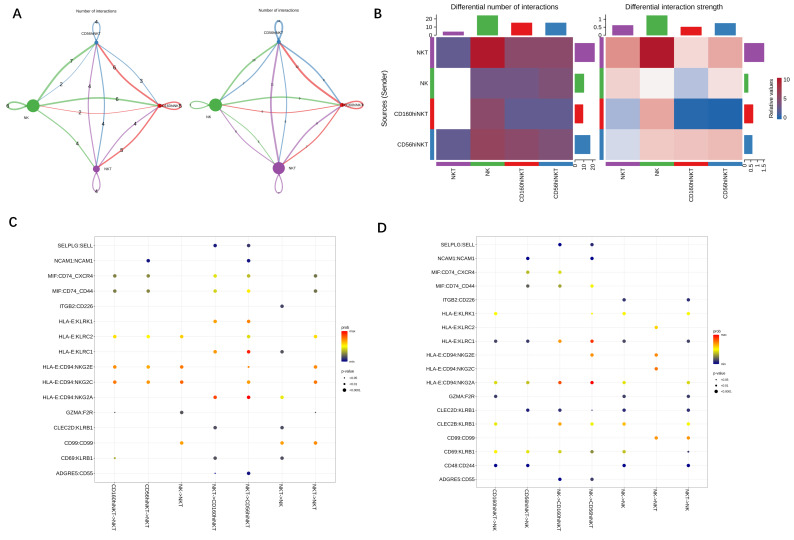
Communications of NKT–NK subsets in patients with CRKP infection. (**A**) Network visualizing inferred differential numbers among circulating NK and NKT cells in healthy individual (**left**) and CRKP colonization patients (**right**). The edges represent the significant ligand–receptor pairs number, and those colored in red (or blue) represent the increased (or decreased) signaling in CRKP infection patients compared with colonization. The direction of arrows represents signal transmission direction from the source to the target. (**B**) Heat map showing the comparison of the interactions numbers (**left**) and strength (**right**) of CRKP infection patients to healthy control. (**C**) Predicted ligand–receptor pairs that contributed to the signaling from NK and NKT cells in CRKP colonization patients. Dot color represents communication probabilities and the size represents computed *p* values. A *p* value is computed from a one-sided permutation test. (**D**) Similar to (**C**), but for CRKP infection patients.

Recent studies on host defense mechanisms have reported that NKT cells can swiftly secrete large amounts of cytokines upon activation by modulating NK cells [11]. How the relationship functions between these two important innate immune cells remains largely unknown. To investigate the modulating effect of NKT cells on the function of NK cells and the relationship with NKT subsets in CRKP infection, we inferred all potential communications by analyzing the expression of ligand–receptor pairs using CellChat. By comparing the number of interactions among NK and NKT cells, we found highly connected pathways appeared during CRKP infection, specifically between the NK and NKT subsets in CRKP colonization patients (Figure 4A,B and Figure S3A,B). To better understand signaling pathways within NK and NKT subsets, we predicted ligand–receptor interaction analysis (Figure 4C,D and Figure S3C,D). We identified HLA-E-NKG2E, HLA-E-NKG2C, HLA-E-CD94, and CLEC2D-KLRB1 ligand–receptor pairs between NK and NKT subsets in CRKP colonization, suggesting that NKT might activate the NK cell by antigen-presenting through specific costimulatory molecules.

## 4. Discussion

In recent years, CRKP infection has become a global public health problem [24,25]. Although great efforts have been made in bacteria variation and drug resistance mechanisms, the development of new antimicrobial drugs is difficult to keep up with the speed of bacterial resistance [26,27]. To solve the problem, this study aimed to investigate the mechanism of CRKP infection from the perspective of host immunity. NKT cells play a pivotal role in the recognition of microbial infections and their subsequent elimination [28,29,30]. Their TCRs induce an explosive release of different cytokines and chemokines that regulate immune cell activation and bridge innate and adaptive immune responses [9]. They are rare cell types composed of multiple subsets; nevertheless, very little is known about the role of NKT subsets in CRKP infection. Through performed scRNA-seq on NK and NKT cells, we were able to capture sufficient cell numbers to define molecular profiles and identify specific defects in CRKP infection.

In this study, we examined the phenotypic and functional variations within circulating NK and NKT subsets with patients under CRKP colonization, infection, and recovery conditions. By analyzing 16,021 individual cells, we identified NKT, CD56hiNKT, and CD160hiNKT populations, and observed NK cells. The results identified a decreased number of circulating NK and CD160hiNKT subsets in CRKP colonization patients. The CD160hiNKT subset was almost absent in patients with CRKP across conditions (Figure 2A). The phenotype of CD160hiNKT cells was largely consistent with previous studies that described traditional NK cells [31,32]. In addition, the marker of T cells, FCGR3A, was also detected in the CD160hiNKT subset, which indicates that it may exhibit antibody-dependent cell-mediated cytotoxicity [33]. The reduction of circulating NK and CD160hiNKT cells from patients with CRKP might contribute to bacterial immune evasion and reflect a suppression state of host immune responses to CRKP.

We observed remarkable growth of the NKT subset abundance after CRKP colonization and increased expression levels of the IL-17 signaling pathway in CD56hiNKT cells from CRKP infection patients, which play an essential role in CRKP clearance (Figure 2A and Figure 3C). The significance of the IL-17 signaling pathway in host defense and disease development has been demonstrated in various infection and autoimmune models [22,34]. One hallmark function of IL-17 is promoting myeloid-driven innate inflammation by induction of chemokines, including CXCL1, CXCL2, and CXCL8 (IL-8) antimicrobial peptides and antimicrobial peptides such as defensins, S100A8, and lipocalin 2; these responses protect the host during the acute microbial invasion [35]. Accordingly, IL-17 responses defend against extracellular fungal and bacterial pathogenic species, including Candida, Cryptococcus, and Staphylococcus, among others [36]. Our results point to the particular importance of IL-17 in immunity to CRKP of CD56hiNKT cells. The underlying mechanisms of the expansion and function of CD56hiNKT cells in CRKP infection deserve future study.

Transcriptomic analysis of innate lymphocytes in the mouse model of endotoxemia suggested that NKT cells depend on the mTOR pathway in NK cells to produce IFN-γ [11]. The role of the mTOR pathway in NK cells has been detected in models of acute myeloid leukemia, hepatoblastoma, liver cancer, and metabolic diseases, but not in bacterial infection [37,38,39,40]. Rapamycin (mTOR) complex 1 (mTORC1) has been reported as a key regulator that promotes glycolytic metabolism in NK cells. The inhibition of mTORC1 could prevent increases in cytolytic and IFN-γ secretion potentials in NK cells stimulated with IL-15. Here, we found an enriched mTORC1 pathway in NK cells from CRKP infection patients, which indicates NK subset function changes contribute to the production of the inflammatory cytokine (Figure 2E). By transcription factor analysis, we detected that EZH2 is the key regulator of the NKT subset in CRKP infection conditions, which could mediate mTOR pathway activation (Figure 3E) [41].

Moreover, the specific interactions between NKT and NK subsets were seen in patients who followed CRKP invasion. The results showed that enhanced interactions between NK and NKT subsets in CRKP infection were mainly caused by increasing HLA-E-NKG2E, HLA-E-NKG2C, HLA-E-CD94, and CLEC2D-KLRB1 expressions (Figure 4A–D). The TCR signal strength influences the polarization of the NKT subset; at the same time, NKT cells can expand, and antigen-specific NKT cell clones can be generated that will assist in the clearance of infection [42,43]. Our analysis suggested that the alteration in intercellular communications involving NKT and NK cells might be a previously underestimated aspect of CRKP infection pathogenesis. It will be valuable to investigate whether NKT cells regulate NK cells by these ligand–receptor (L-R) pairs in CRKP infection.

In summary, in this study NKT cells within a healthy individual and patients in CRKP colonization, infection, and recovery conditions displayed an exquisite division of labor. The expansion, contraction, or alteration of NKT subsets in pathological settings will guide the development of appropriate therapies.

## Data Availability

Not applicable.

## Supplementary Materials

The following supporting information can be downloaded at: https://www.mdpi.com/article/10.3390/jpm12122025/s1, Figure S1: Basic information of the scRNA-seq data. (A) Number of read counts, cell number (B), UMI, unique molecular identifier, number (C), gene number (D), and percentage of mitochondrial gene (E), detected in each cluster; Figure S2: Analysis of NKT subsets across conditions, related to Figure 3. The top 10 enriched biological processes of genes in cluster 1 (A), cluster 2 (B), cluster 3 (C), and cluster 6 (D) defined in Figure 3A as revealed by Reactome analysis; Figure S3: Interaction between NK and NKT cell types across conditions, related to Figure 4. (A), Network visualizing inferred differential number among circulating NK and NKT cells in CRKP infection (left) and (B) recovery patients (right). The edges represent the significant ligand–receptor pairs number and which colored in red (or blue) represent the increased (or decreased) signaling in CRKP infection patients compared with colonization. The direction of arrows represents signal transmission direction from the source to the target. (C), Predicted ligand-receptor pairs which contributed to the signaling from NK and NKT cells in healthy control. Dot color represent communication probabilities and the size represents computed *p*-values. A *p* value is computed from a one-sided permutation test. (D) Similar to (C), but for CRKP recovery patients.
