# Supplementary material for: Diversity of Circulating NKT Cells in Defense against Carbapenem-Resistant Klebsiella Pneumoniae Infection
Source: J Pers Med. 2022 Dec 7;12(12):2025. doi: 10.3390/jpm12122025 (PMC9783671; doi:10.3390/jpm12122025)
Supplement: Supplementary file 1 [file jpm-12-02025-s001.zip › Supplementary figure legends.pdf]

Fig. S1: Basic information of the scRNA-seq data.

(A) Number of read counts, cell number (B), UMI, unique molecular identifier, number (C), gene number (D), and percentage of mitochondrial gene (E) detected in each cluster.

Fig. S2: Analysis of NKT subsets across conditions, related to Fig. 3.

The top 10 enriched biological processes of genes in cluster 1 (A), cluster 2 (B), cluster 3 (C), and cluster 6 (D) defined in Fig. 3A as revealed by Reactome analysis.

Fig. S3: Interaction between NK and NKT cell types across conditions, related to Fig. 4.

(A) Network visualizing inferred differential number among circulating NK and NKT cells in CRKP infection (left) and recovery patients (right). The edges represent the significant ligand–receptor pairs number and which colored in red (or blue) represent the increased (or decreased) signaling in CRKP infection patients compared with colonization. The direction of arrows represents signal transmission direction from the source to the target. (B) Predicted ligand-receptor pairs which contributed to the signaling from NK and NKT cells in healthy control. Dot color represent communication probabilities and the size represents computed *p*-values. A *p* value is computed from a one-sided permutation test. (D) Similar to C, but for CRKP recovery patients.
