# Supplementary figures and images for: Diversity of Circulating NKT Cells in Defense against Carbapenem-Resistant Klebsiella Pneumoniae Infection
Source: J Pers Med. 2022 Dec 7;12(12):2025. doi: 10.3390/jpm12122025 (PMC9783671; doi:10.3390/jpm12122025)

**A**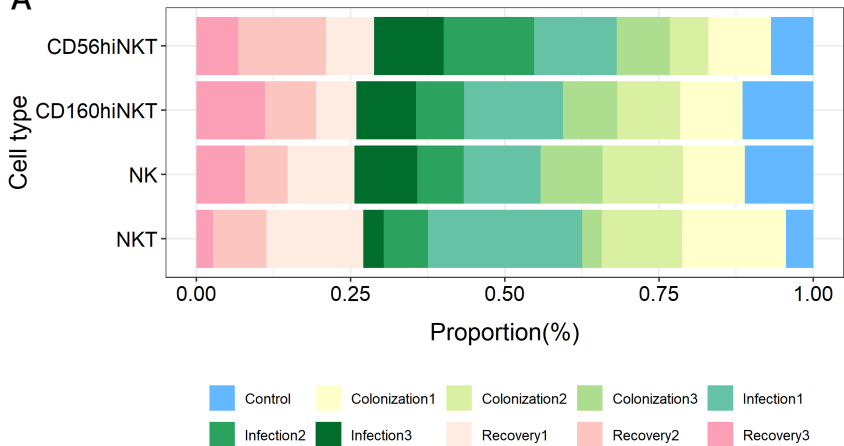**B**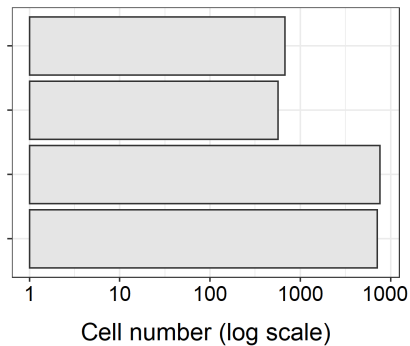**C**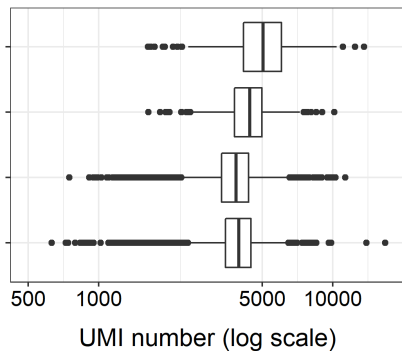**D**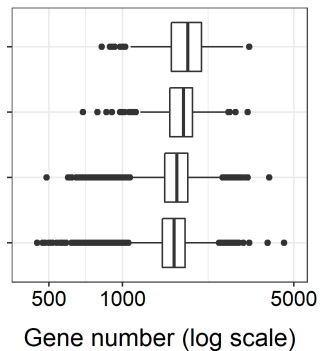**E**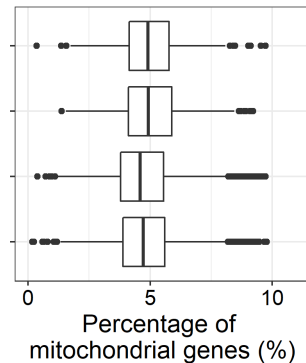

Supplement: Supplementary file 1 [file jpm-12-02025-s001.zip › Figure S1.pdf]

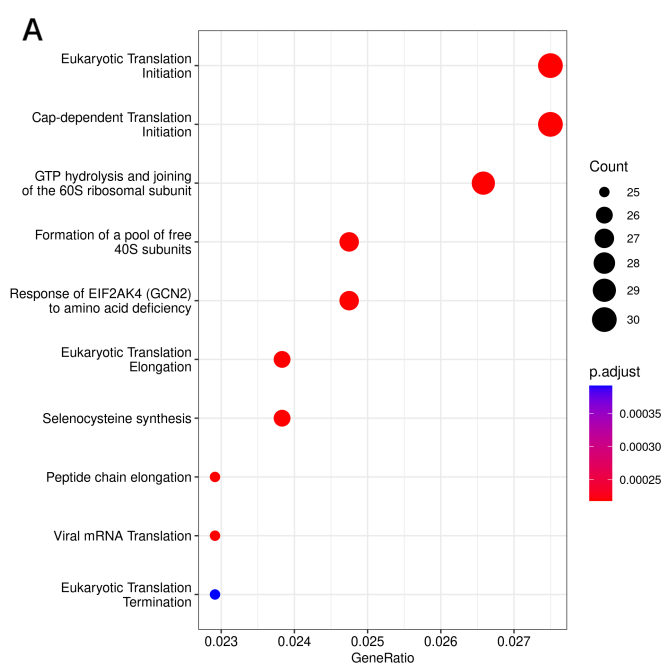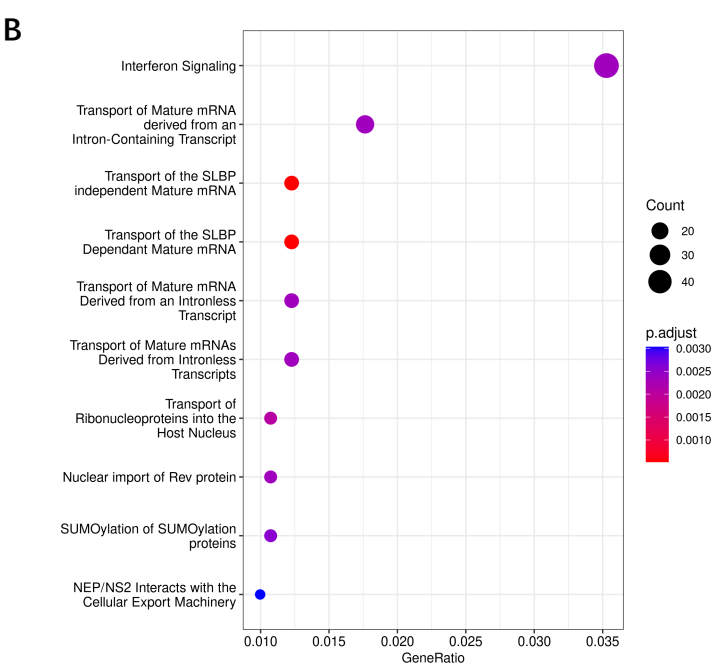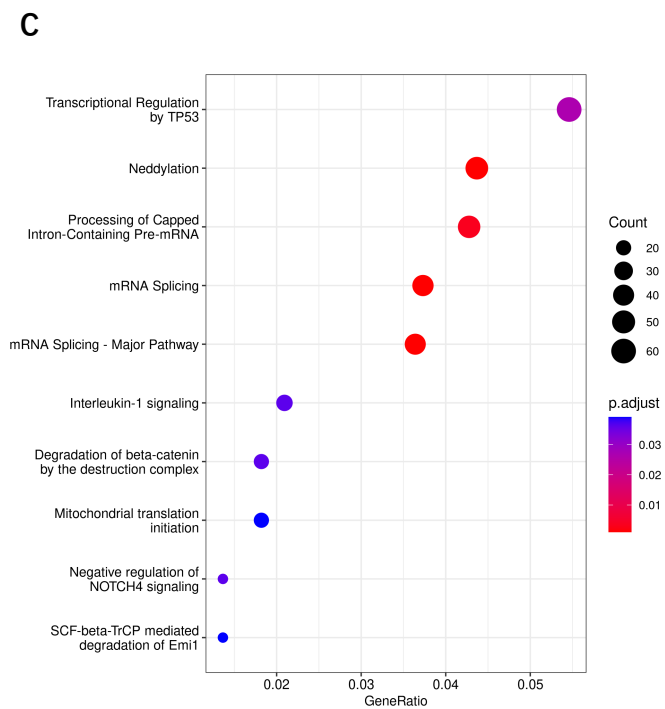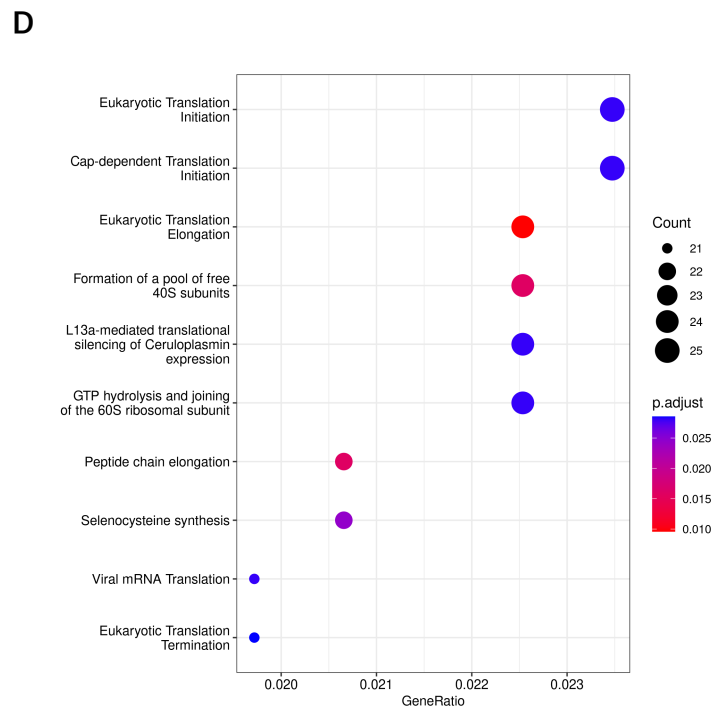

Supplement: Supplementary file 1 [file jpm-12-02025-s001.zip › Figure S2.pdf]

**A**

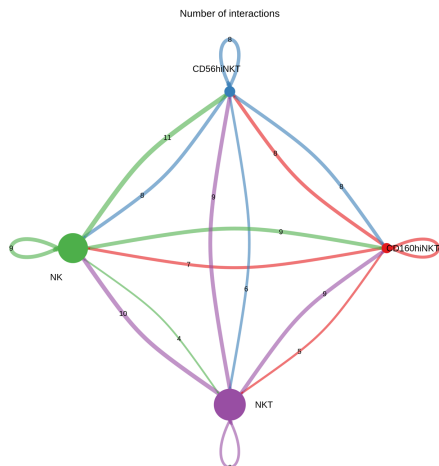

**B**

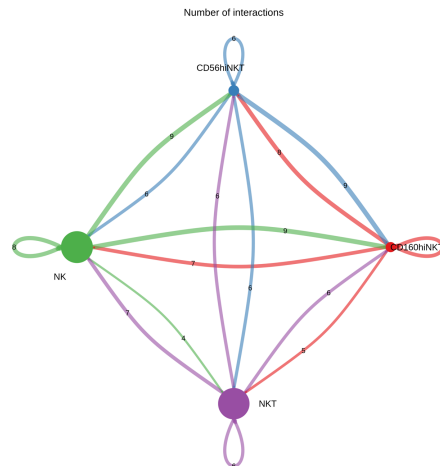

**C**

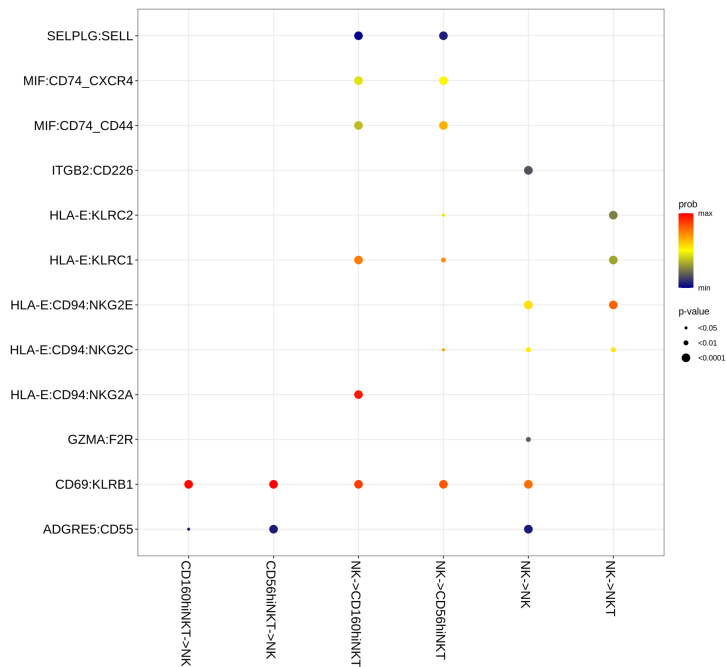

**D**

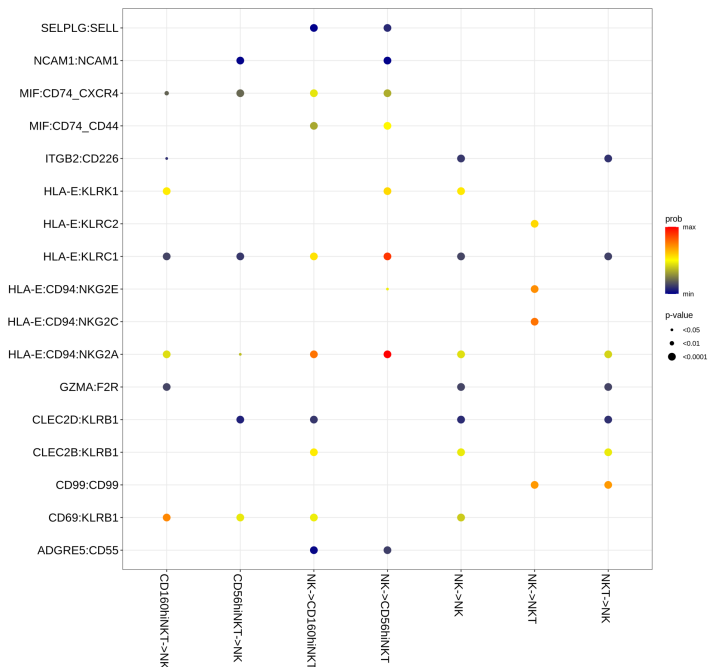

Supplement: Supplementary file 1 [file jpm-12-02025-s001.zip › Figure S3.pdf]
